# Supplementary material for: Cyanidin-3-O-glucoside Contributes to Leaf Color Change by Regulating Two bHLH Transcription Factors in Phoebe bournei
Source: Int J Mol Sci. 2023 Feb 14;24(4):3829. doi: 10.3390/ijms24043829 (PMC9960835; doi:10.3390/ijms24043829)
Supplement: Supplementary file 1 [file ijms-24-03829-s001.zip › ijms-2189297-supplementary.pdf]

Figure S1. The transcription factors enriched in Sub Class1 and Sub Class2;

Figure S2. The phylo-genetic tree of the bHLH transcription factors. AtbHLH: AT5G41315.1, AT1G63650.1; AcbHLH: ranscription factor bHLH42 Actinidia chinensis; CmbHLH: bHLH transcription factor 2 Chry-santhemum x morifolium; PtrbHLH : Potri.001G103600, Potri.003G128000, Potri.005G208600;

Figure S3. Analysis of cis-acting elements of PbbHLH1 and PbbHLH2 promoters. (A) Analysis of the cis-acting element of the PbbHLH1 promoter; (B) Analysis of the cis-acting element of the PbbHLH2 promoter;

Table S1. Primer sequences for molecular cloning of PbbHLH1 and PbbHLH2;

Table S2. Primer sequences for qPCR analysis;

Table S3. Total identified metabolites in S1, S2 and S3;

Table S4. VIP value of all anthocyanins;

Table S5. RNA-seq data;

Table S6. Pathway enrichment of DEGs in three developmental stages;

Table S7. Expression of genes in anthocyanidins biosynthesis pathways;

Table S8. Correlation coefficient between TFs and Metabolites.
