# Supplementary figures and images for: Cyanidin-3-O-glucoside Contributes to Leaf Color Change by Regulating Two bHLH Transcription Factors in Phoebe bournei
Source: Int J Mol Sci. 2023 Feb 14;24(4):3829. doi: 10.3390/ijms24043829 (PMC9960835; doi:10.3390/ijms24043829)

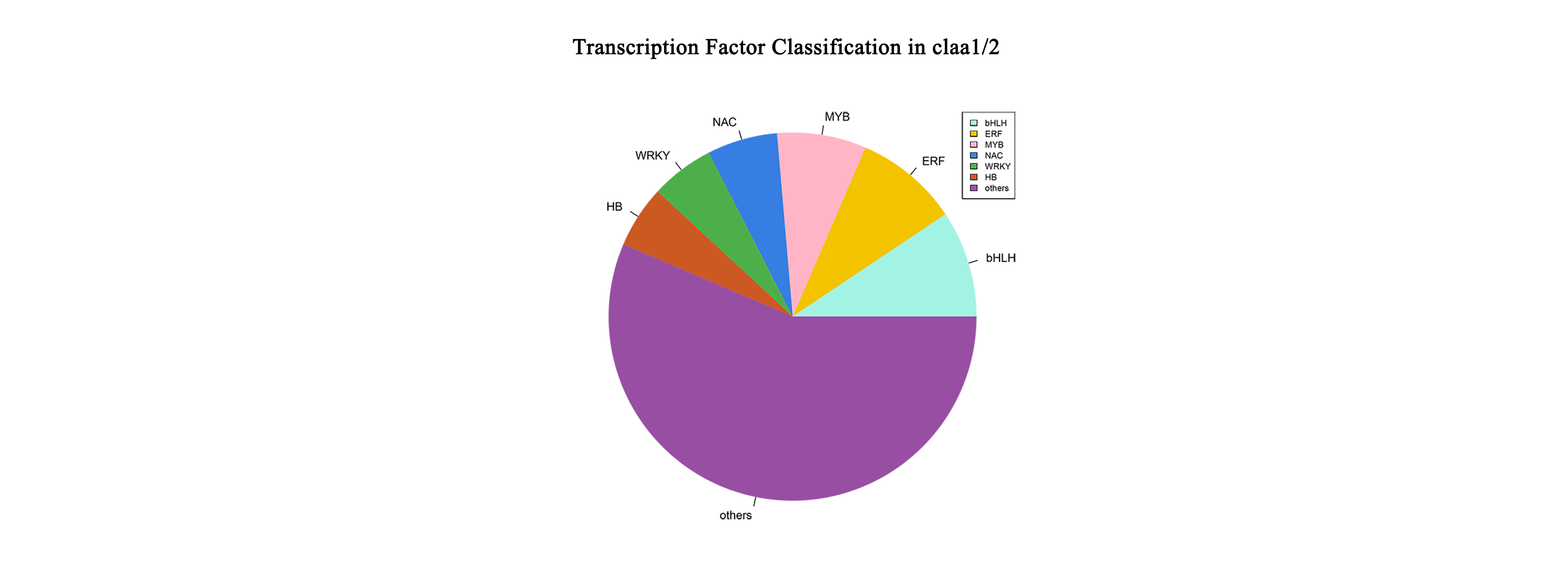

Supplement: Supplementary file 1 [file ijms-24-03829-s001.zip › Figure S1 The transcription factors enriched in Sub Class1 and Sub Class2.tif]

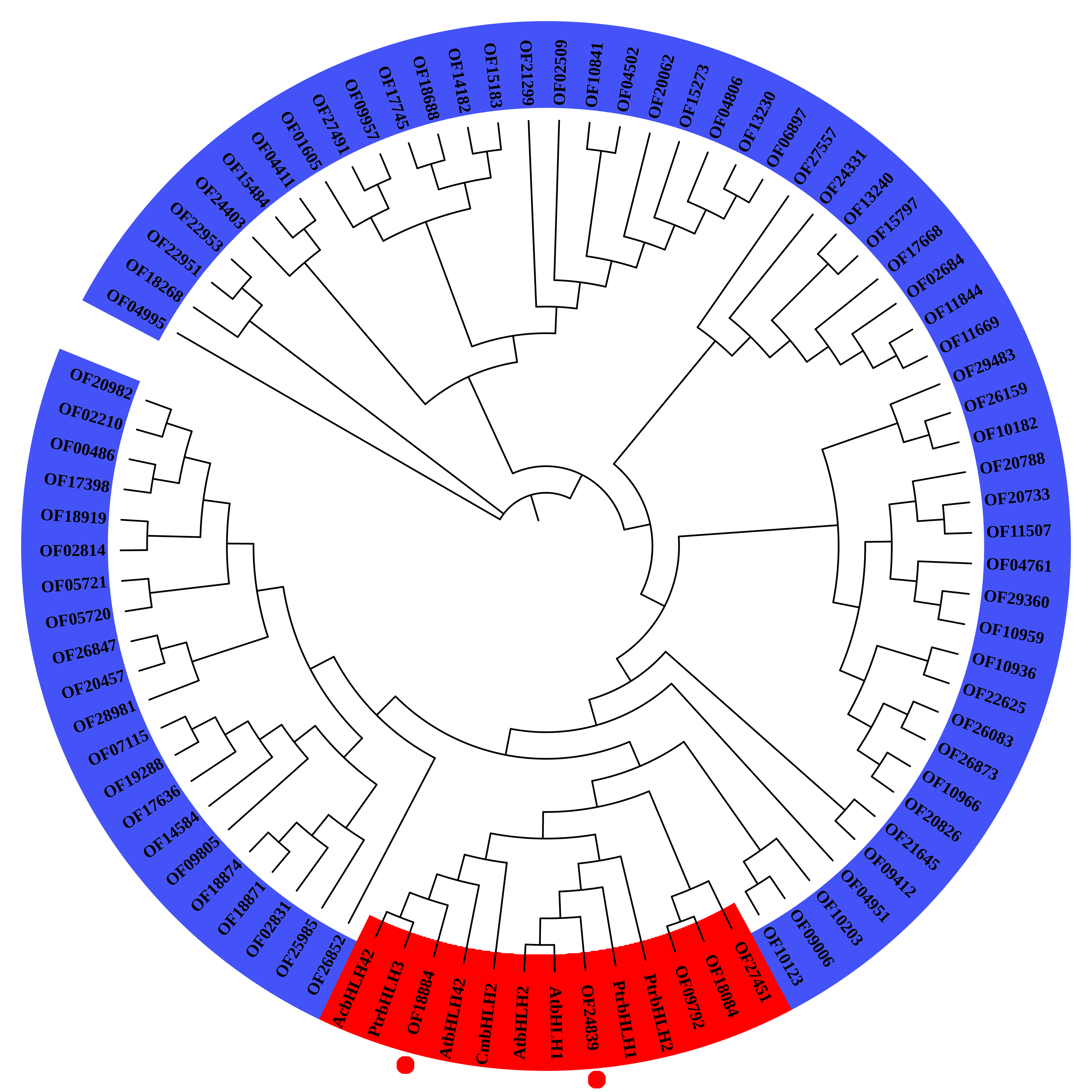

Supplement: Supplementary file 1 [file ijms-24-03829-s001.zip › Figure S2 The phylogenetic tree of the bHLH transcription factors..tif]
